# Inability to Ventilate Due to a Sudden Intraoperative Tension Hydrothorax

**DOI:** 10.7759/cureus.112674

**Published:** 2026-07-14

**Authors:** Gregory Smith, Ashwin Jitheesh, Austin Le, Galina Leyvi, Pradeep Balasubramaniam

**Affiliations:** 1 Medicine, Touro College of Osteopathic Medicine, New York, USA; 2 Anesthesiology, New York Medical College at St. Mary's General Hospital, Passaic, USA; 3 Anesthesiology, New York Medical College at St. Mary's General Hospital and St. Clare's Health, Passaic, USA

**Keywords:** chest tube drainage, intra-operative cardiac arrest, pleural effusion, tension hydrothorax, ventilation failure

## Abstract

Intraoperative ventilatory failure is most often attributed to airway or equipment problems, but rare pleural complications can produce abrupt obstructive physiology. We report an 85-year-old woman with severe mitral and aortic stenosis, end-stage renal disease (ESRD) on hemodialysis, and recurrent pleural effusions undergoing video-assisted thoracoscopic surgery (VATS) with bilateral PleurX catheter placement who developed sudden ventilation failure immediately after left-sided catheter placement. Despite prompt airway reassessment and emergent pleural decompression, she progressed to cardiopulmonary arrest with echocardiographic findings consistent with profound hemodynamic compromise. This case highlights tension hydrothorax as a critical diagnostic consideration when unexplained ventilatory failure follows pleural instrumentation, particularly in patients with limited preload reserve.

## Introduction

A tension hydrothorax is a rare, life-threatening complication that requires urgent recognition and treatment [1]. This feared catastrophic event is characterized as a massive pleural effusion that, if untreated and unrecognized, can lead to cardiac tamponade physiology and cardiorespiratory collapse [1-3].

It is well documented that massive pleural effusions compromise cardiopulmonary function; however, there is a paucity of literature highlighting the ventilation challenges and perioperative management strategies associated with this rare pathology [1-3]. Large pleural effusions may shift mediastinal structures, increase intrathoracic pressure, impair venous return, and reduce cardiac output and diastolic filling [1-3]. Moreover, rapidly evolving pleural fluid accumulation may cause ipsilateral lung compression, reducing alveolar ventilation and inducing intrapulmonary shunt, thereby creating ventilation-perfusion (V/Q) mismatch [4].

The incidence of this catastrophic event is difficult to ascertain, as it has been described primarily in isolated reports rather than larger cohort studies [1-3,5-9]. Reported manifestations include tamponade physiology, rising airway pressures, falling pulmonary compliance, hypoxemia, and possible loss of unilateral ventilation [1,8-10]. In mechanically ventilated patients, evolving tension physiology may initially present as unexplained ventilatory failure with rising airway pressures and worsening compliance, potentially delaying recognition and decompression [6-9].

Prior intraoperative reports have described tension hydrothorax arising from transdiaphragmatic fluid migration during abdominal and pelvic procedures; however, its occurrence during direct pleural instrumentation in a patient with combined fixed-outflow valvular obstruction and end-stage renal disease has not been previously described [6-9]. Here, we present a case of sudden intraoperative loss of ventilation and subsequent cardiorespiratory collapse most consistent with acute tension hydrothorax following pleural catheter placement in an 85-year-old mechanically ventilated patient. This case highlights the diagnostic challenge posed by evolving tension physiology in patients with markedly limited cardiopulmonary reserve.

## Case presentation

An 85-year-old woman presented for video-assisted thoracoscopic surgery (VATS) with planned bilateral PleurX^TM^ catheter placement for management of recurrent pleural effusions. Her medical history included systolic and diastolic heart failure, severe mitral and aortic stenosis, end-stage renal disease (ESRD) requiring hemodialysis three times weekly, and a cerebrovascular accident nine years prior with residual left-sided weakness.

Recent clinical course

Our patient was a nursing home resident who developed sepsis attributed to right upper lobe (RUL) pneumonia with respiratory failure requiring endotracheal intubation. An extubation attempt was unsuccessful, necessitating re-intubation and subsequent tracheostomy. After treatment of left-sided pneumonia and discharge to the nursing home on a tracheostomy collar, worsening mentation was noted, and she was transferred to a long-term acute care (LTAC) facility for respiratory weaning.

Preoperative evaluation

A previous cardiology note from 2016 described an echocardiogram revealing preserved left ventricular systolic function (ejection fraction 65-70%), mild mitral annular calcification with trace mitral regurgitation, and aortic valve sclerosis with trivial regurgitation. Estimated systolic pulmonary artery pressure was 40.7 mmHg, and grade I left ventricular diastolic dysfunction (impaired relaxation) was noted.

**Figure 1:**
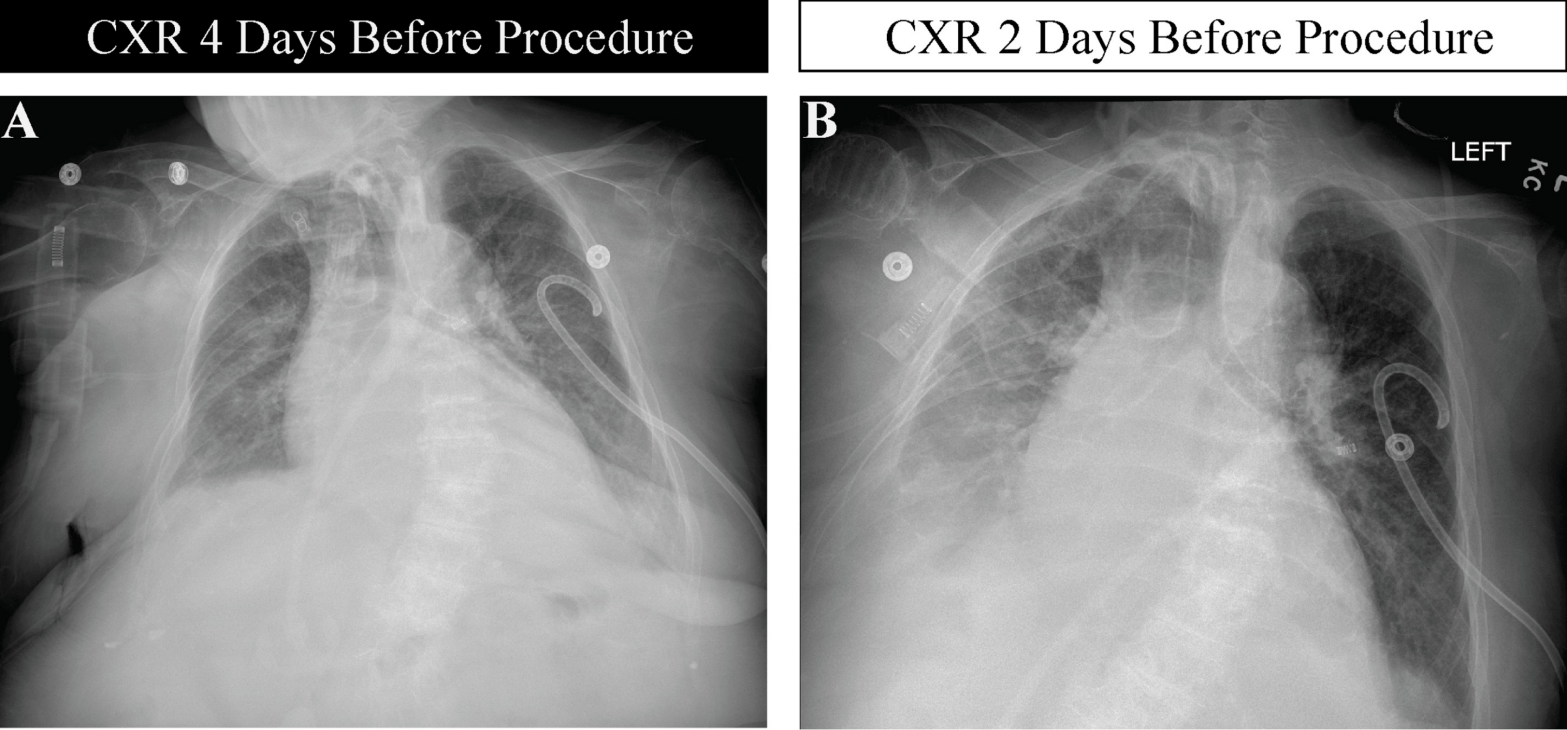
Chest radiographs obtained four and two days prior to the procedure Portable anteroposterior (AP) chest radiographs obtained four days (A) and two days (B) prior to the procedure. The radiographs demonstrate post-tracheostomy status with the tracheostomy tube tip projecting approximately 5.5 cm above the carina. Both images demonstrate moderate cardiomegaly with bilateral pulmonary vascular congestion, a moderate right pleural effusion, and a mild left pleural effusion with adjacent atelectasis. A left pleural pigtail catheter is visualized. Compared with the prior radiograph (A), radiograph B demonstrates interval progression of the right pleural effusion. CXR - chest X-ray

Immediately prior to surgery in 2025, a cardiology note documented an echocardiogram demonstrating a reduced ejection fraction of 45-50% in the setting of known severe mitral and aortic stenosis. Chest radiography obtained two and four days preoperatively showed moderate cardiomegaly with bilateral pulmonary vascular congestion, a moderate right pleural effusion, and a mild left pleural effusion with adjacent atelectasis; no pneumothorax was present (Figure 1). The right pleural effusion appears to have progressed from day four to day two. Tracheostomy evaluation demonstrated the tube tip approximately 5.5 cm above the carina.

Airway assessment was notable for poor dentition, limited cervical range of motion, and an indeterminate Mallampati classification. The patient was classified as ASA physical status IV. A left-sided chest tube was removed by the surgeon immediately prior to induction, draining approximately 700 mL of pleural fluid. Inhalational induction with sevoflurane was planned with neuromuscular blockade using rocuronium (20 mg).

Intraoperative course

The patient was positioned in the right posterolateral thoracotomy position with standard American Society of Anesthesiologists (ASA) monitors. Intercostal nerve blocks were administered at three sites along the left thoracic wall using a mixture of 0.5% bupivacaine and liposomal bupivacaine (Exparel). A PleurX catheter (Becton Dickinson, Franklin Lakes, New Jersey) was tunneled through anterior and posterior exit sites. Baseline peak inspiratory pressure (PIP) was 25 cmH_2_O with a tidal volume of 305 mL, SpO_2_ of 99%, and EtCO_2_ of 43 mmHg. By 12:45, during catheter placement, PIP had risen to 30 cmH_2_O and tidal volume had fallen to 68 mL with EtCO_2_ dropping to zero, suggesting early ventilatory compromise developing during pleural instrumentation. After suctioning through the tracheostomy, a guidewire was introduced using the Seldinger technique, and catheter placement was confirmed visually via thoracoscopy. The catheter was connected to a PleurX drainage bottle and Pleura-Vac suction.

**Figure 2:**
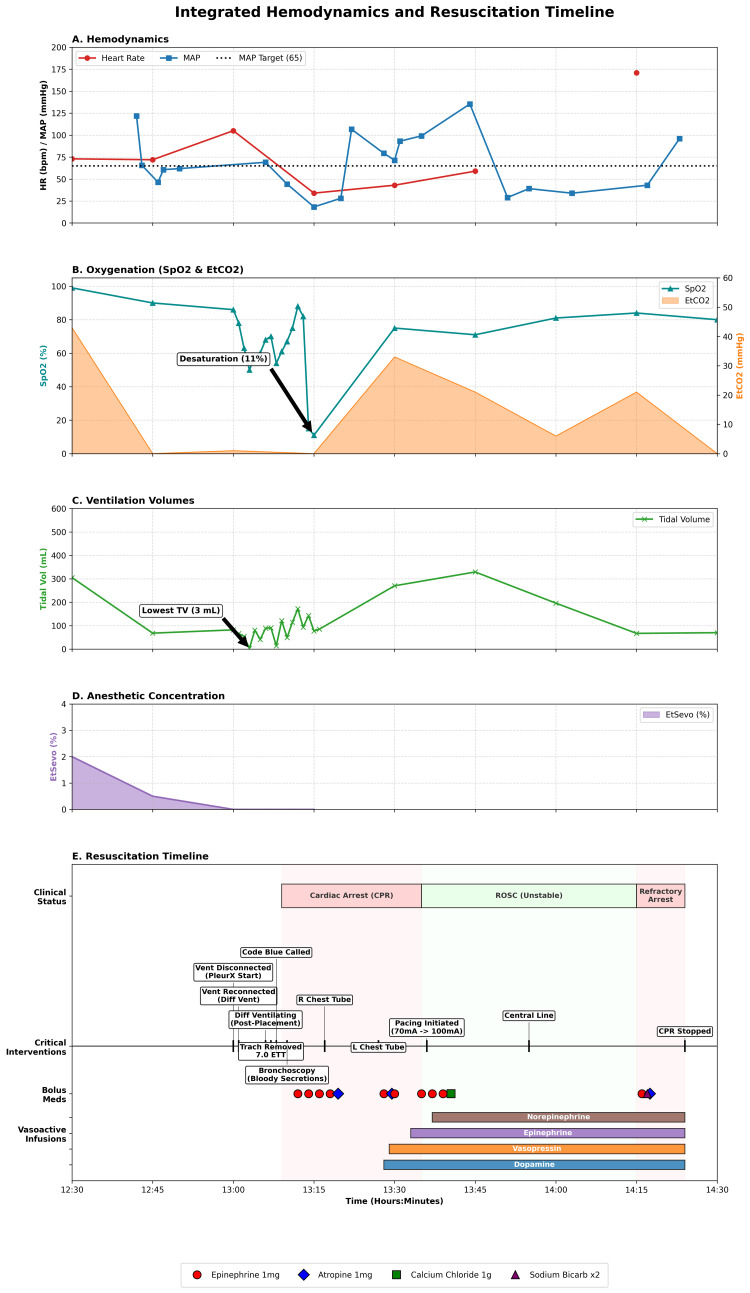
Integrated anesthesia record timeline during PleurX catheter placement (A) Heart rate and mean arterial pressure (MAP) with MAP target (65 mmHg). (B) Oxygenation and ventilation trends, SpO₂ and end-tidal CO₂ (EtCO₂), highlighting the nadir desaturation. (C) Observed tidal volumes (Vt), including the lowest recorded Vt. (D) End-tidal sevoflurane (EtSevo) concentration. (E) Resuscitation timeline with key procedural and ACLS events. At 13:00, the ventilator circuit was temporarily disconnected from the tracheostomy to facilitate catheter placement, and oxygen saturation declined during this interval. After reconnection at 13:01, small tidal volumes and persistent difficulty ventilating were documented, with progressive deterioration during continued pleural instrumentation and PleurX placement at 13:06. These changes were accompanied by falling SpO₂ with contemporaneous EtCO₂ changes, consistent with worsening ventilation and oxygenation preceding cardiopulmonary resuscitation.

At 13:00, the ventilator circuit was temporarily disconnected from the tracheostomy to assist with catheter placement, at which point SpO_2_ was 86%. Upon reconnection at 13:01, difficulty ventilating with small tidal volumes was immediately documented with SpO_2_ falling to 78% despite reconnection. At 13:06, ventilatory difficulty worsened immediately following PleurX catheter placement, with SpO_2_ at 68% and tidal volume of 89 mL. At 13:07, the tracheostomy was removed, and orotracheal intubation was performed with a 7.0 mm endotracheal tube. A code blue was called at 13:08 and chest compressions were initiated at 13:09. At 13:10, endotracheal tube placement was confirmed by bronchoscopy with copious bloody secretions suctioned from the oropharynx. Despite a secured and bronchoscopically confirmed airway, PIP reached a peak of 45 cmH_2_O with a tidal volume of 73 mL and SpO_2_ declining to a nadir of 11% at 13:15, consistent with severely reduced respiratory system compliance rather than isolated airway obstruction and indicating a compliance-based rather than resistance-based mechanism of ventilatory failure (Figure 2).

**Figure 3:**
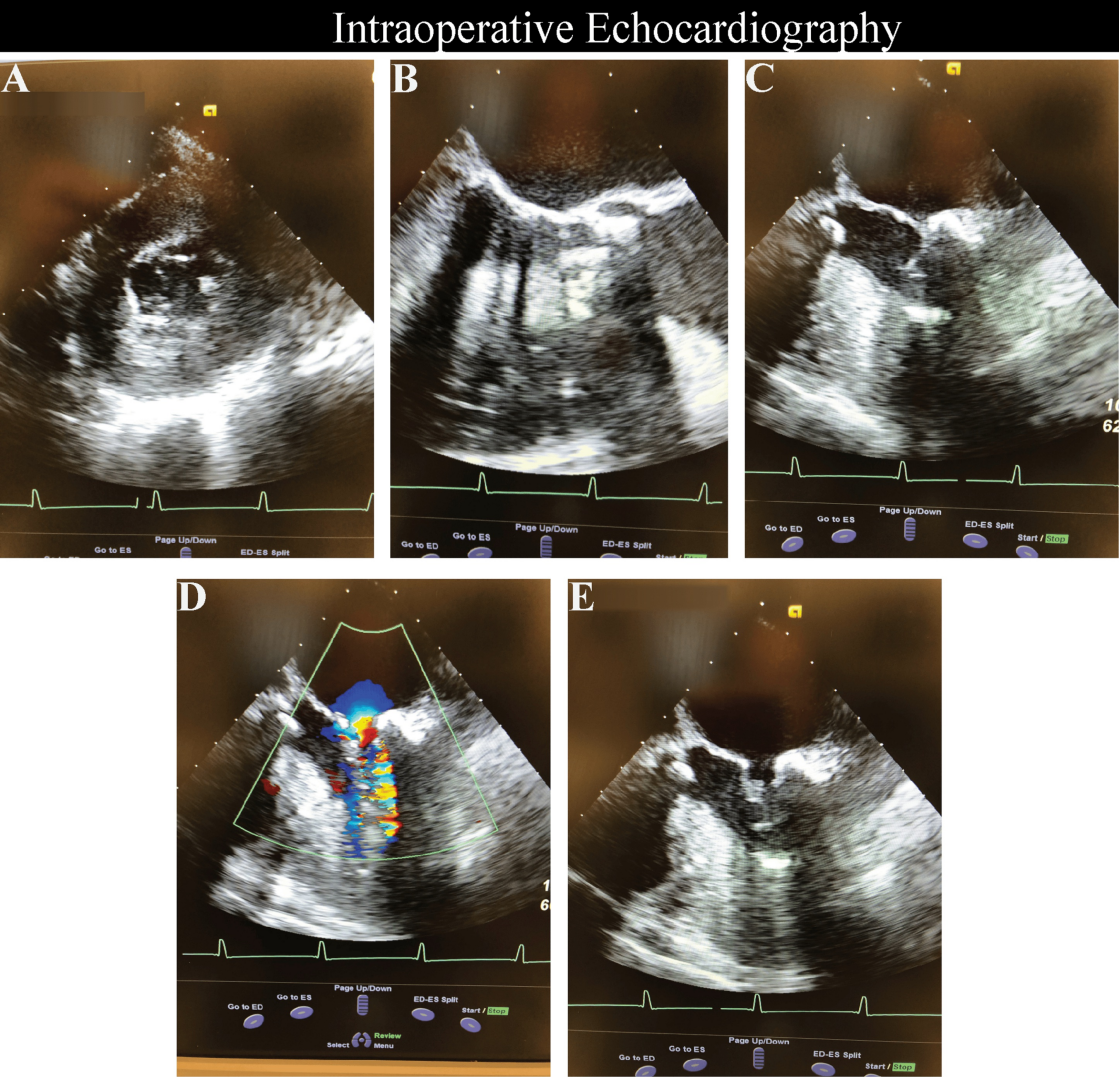
Intraoperative transesophageal echocardiography (TEE) demonstrating severe concentric left ventricular (LV) hypertrophy with a small, underfilled LV cavity and combined aortic and mitral stenosis physiology (A) Marked concentric left ventricular (LV) hypertrophy with a small LV cavity. (B) Severely stenotic aortic valve. (C) Systolic anterior motion of the mitral valve anterior leaflet during systole, suggestive of dynamic obstruction in a small-cavity, hypertrophied ventricle. (D) Color Doppler demonstrating prominent intracavitary flow acceleration/turbulence consistent with dynamic obstruction in an underloaded LV. (E) Thickened mitral valve leaflets with restricted opening during diastole, consistent with mitral stenosis.

Cardiopulmonary resuscitation was initiated with ongoing manual ventilation (Figure 2). At 13:17, a 32-French chest tube was inserted into the right pleural space, draining approximately 1050 mL of serosanguineous fluid under Pleura-Vac suction at -40 cm H₂O. At 13:25, a transesophageal echocardiography (TEE) probe was placed by a cardiac anesthesiologist and demonstrated global left ventricular akinesis. External pacing produced only transient mechanical activity without sustained perfusion (Figure 3).

At 13:27, a second 32-French chest tube was placed on the left, yielding approximately 500 mL of serosanguineous fluid. A Cordis central venous catheter was inserted into the left internal jugular vein for hemodynamic monitoring and rapid infusion. Despite approximately one hour of pharmacologic resuscitation and advanced cardiac life support (ACLS) measures, the patient remained in asystole with persistent left ventricular akinesis on TEE. After discussion with the family, death was pronounced.

## Discussion

This case describes a cardiopulmonary collapse that occurred in a patient whose reserve was constrained by fixed or relatively fixed forward flow (mitral stenosis and aortic stenosis) and limited compensatory capacity from end-stage renal disease (ESRD). Additionally, our case expands upon prior reports of tension hydrothorax by illustrating a perioperative presentation in a profoundly preload-limited circulation, where modest intrathoracic pressure changes may precipitate rapid decompensation.

In mitral stenosis, left ventricular filling is limited by restricted transmitral flow, making cardiac output sensitive to tachycardia, loss of atrial contribution, and increases in pulmonary vascular pressures [11-13]. In aortic stenosis, stroke volume is constrained by outflow obstruction, so hypotension and abrupt reductions in preload are poorly tolerated, and coronary perfusion can fall quickly [14,15]. ESRD adds further fragility through chronic volume shifts, uremic cardiomyopathy, anemia, and limited ability to buffer acute hemodynamic stress [16,17].

Within that narrow physiologic window, an abrupt pleural process can become hemodynamically dominant [1-3,5-10]. Large pleural fluid collections can impair ventilation and promote intrathoracic tension physiology by shifting the mediastinum, reducing venous return, and limiting right-sided filling, which can produce a clinical picture consistent with obstructive shock [1-3,5-10]. Although definitive pleural pressure measurements were not available, the rapidity of decompensation, refractory ventilation despite a patent airway, obstructive physiology on intraoperative echocardiography (when present), and large-volume pleural drainage temporally associated with partial physiologic improvement collectively support tension hydrothorax as the most coherent explanation [1-3,6-10]. Prior reports describe tension hydrothorax presenting with mediastinal shift, ventilatory failure, and shock [2,3,5-9]. 

Several competing diagnoses deserve consideration when ventilation and hemodynamics collapse abruptly in a tracheostomized patient: mucus plugging or tracheostomy displacement, pneumothorax, hemothorax, and re-expansion pulmonary edema. Each was considered systematically in the context of the specific clinical findings present in this case.

Pneumothorax was considered given the lateral decubitus positioning and rising airway pressures, while hemothorax was considered given the bloody secretions suctioned from the oropharynx following intubation. However, insertion of a 32-French chest tube into the right pleural space yielded approximately 1050 mL of fluid and a second chest tube on the left yielded approximately 500 mL, with both sides draining serosanguineous fluid rather than frank blood or air, arguing against both pneumothorax and hemothorax as primary etiologies [18].

Re-expansion pulmonary edema generally evolves after drainage rather than at the moment of sudden obstructive collapse and is characterized by hypoxemia with pulmonary edema physiology driven by inflammatory microvascular permeability rather than primary venous return limitation [19]. In this case, ventilatory failure and hemodynamic collapse preceded pleural drainage, and echocardiographic findings were consistent with obstructive rather than pulmonary edema physiology, making this diagnosis unlikely.

Airway-related etiologies were considered given abrupt ventilatory failure with rising airway pressures and copious bloody secretions requiring repeated suctioning; however, persistent ventilatory failure despite patient repositioning, orotracheal intubation, and bronchoscopic confirmation of tube position collectively argued against a primary airway process as the sole etiology [20]. The persistence of elevated airway pressures and minimal tidal volumes despite a secured and confirmed airway was a critical inflection point suggesting an alternative intrathoracic mechanism.

In this context, the combination of sudden ventilatory failure immediately following pleural catheter placement, refractory ventilation despite a secured and bronchoscopically confirmed airway, large volume pleural drainage from both sides, and TEE findings of a markedly underfilled hypertrophied left ventricle with dynamic obstruction aligned more closely with tension physiology than with these alternatives [1-3, 6-10]. Notably, right-sided chest tube drainage of approximately 1050 mL was temporally associated with partial physiologic improvement, providing direct clinical evidence linking pleural decompression to hemodynamic response and supporting tension hydrothorax as the primary mechanism.

For patients with preload dependence or fixed outflow, pleural interventions and intrathoracic pressure changes can precipitate rapid transition from compensated respiratory compromise to obstructive shock within minutes [1-3,5,13,17]. When abrupt decompensation follows pleural instrumentation or occurs in the setting of a large effusion, early bedside ultrasound and/or transesophageal echocardiography can help distinguish obstructive physiology from primary pump failure and guide immediate decompression [1,2,5-9]. Additionally, anesthetic technique selection warrants careful consideration in this population; the hemodynamic consequences of positive pressure mechanical ventilation, including reduced venous return and altered intrathoracic pressure dynamics, may further narrow the already limited physiologic window in patients with fixed-outflow valvular obstruction and end-stage renal disease, and regional anesthetic approaches merit prospective consideration where procedural requirements permit.

## Conclusions

This case describes a presentation most consistent with tension hydrothorax precipitating abrupt cardiopulmonary collapse during pleural intervention, particularly in a patient with severely limited reserve from combined valvular stenosis and ESRD. In such patients, small changes in intrathoracic pressure and venous return can rapidly translate into critical reductions in forward flow and oxygenation. Here, the temporal association between pleural catheter placement, sudden loss of effective ventilation, large-volume bilateral pleural drainage, and echocardiographic evidence of a markedly underfilled obstructed ventricle collectively supports tension physiology as the most clinically coherent unifying explanation, though definitive confirmation was not possible in this emergent setting. The diagnosis remained clinical, based on the convergence of circumstantial evidence rather than direct pleural pressure measurement, and alternative etiologies cannot be entirely excluded. Although pleural decompression was performed, the clinical trajectory suggests that the physiologic threshold for recovery had already been exceeded. These findings emphasize the diagnostic challenge posed by sudden ventilatory failure after pleural instrumentation and support maintaining a high index of suspicion for tension hydrothorax, particularly when coexisting valvular, renal, and pulmonary disease markedly narrows hemodynamic resilience under anesthesia.
